# Aid cut, lives lost: estimating the impact of USAID’s withdrawal on maternal mortality in six African countries

**DOI:** 10.1093/heapol/czag034

**Published:** 2026-03-10

**Authors:** Matthew Cummins

**Affiliations:** United Nations Population Fund, West and Central Africa Regional Office, 8 Route des Almadies, Dakar 10200, Senegal

**Keywords:** maternal mortality, maternal health, primary healthcare expenditure, official development assistance, foreign aid, West and Central Africa

## Abstract

In January 2025, the US government suspended and subsequently terminated the majority of United States Agency for International Development (USAID) programs. This study estimates the impact of that decision on maternal mortality in six highly vulnerable countries in West and Central Africa: Burkina Faso, Central African Republic, Chad, Mali, Niger, and Nigeria. Using a deterministic model grounded in regional health expenditure elasticities, the analysis projects how the sudden withdrawal of foreign aid affects health spending among populations in humanitarian need, under the assumption that no immediate domestic or external financing substitutes for the lost resources, and the resulting changes in maternal mortality ratios (deaths per 100 000 live births). The results indicate that the funding cuts could cause maternal deaths to increase by 45%, on average, among populations in need. This increase is estimated relative to a baseline of approximately 2900 maternal deaths predicted in 2025, yielding approximately 1000 additional deaths across the countries within a single year. The magnitude of impact varies, with Niger experiencing the largest proportional increase (over 90%) and Nigeria the largest absolute increase (more than 300 additional deaths). Sensitivity analyses confirm that the results are robust to alternative elasticity scenarios. The findings illustrate the degree to which maternal health outcomes in fragile settings are sensitive to financing discontinuities. The results are presented as conditional estimates and are intended to inform ongoing discussions on health financing sustainability, transition planning, and risk mitigation.

Key message

- Sudden cuts to USAID health funding in six West and Central African countries are projected to increase maternal mortality by approximately 45% among populations in humanitarian need in 2025, corresponding to over 1000 additional deaths under a no-substitution scenario.

Key findings and major points Sudden cuts to USAID funding to health systems in six West and Central African countries are projected to increase maternal mortality by approximately 45% among populations in humanitarian need in 2025, corresponding to over 1000 additional deaths under a no-substitution scenario.

The impacts are uneven: Niger is projected to experience the largest proportional rise in maternal mortality (over 90%) and Nigeria the highest absolute increase in deaths (more than 300). Countries with weaker domestic health budgets are most vulnerable to donor withdrawal.

This analysis provides the first quantified estimates of maternal mortality shocks from abrupt aid cuts in fragile health systems.

## Introduction

Official development assistance (ODA) remains a critical source of financing for public health services in low-income countries. Historically, the United States (US) has been the largest bilateral contributor, accounting for more than 40% of all international health assistance from donor governments (Oum et al. 2025). On 20 January 2025, US President Donald Trump issued an Executive Order mandating a 90-day pause and assessment of American foreign aid, effectively suspending most overseas programs, including for public health (US White House 2025). In March 2025, this pause was made permanent through the cancelation of more than 80% of programs managed by the United States Agency for International Development (USAID) (Knickmeyer 2025). In several low-income and crisis-affected settings, USAID funding represented a substantial share of resources supporting basic health services, including maternal health.

This article estimates the potential increase in maternal deaths among highly vulnerable populations in six countries in West and Central Africa following the sudden end of USAID funding. It develops a deterministic model linking changes in health expenditure to maternal mortality ratios (MMRs, deaths per 100 000 live births). The countries under study—Burkina Faso, Central African Republic, Chad, Mali, Niger, and Nigeria—have some of the highest MMRs globally (World Health Organization or WHO 2025a) and face overlapping humanitarian, political, and macroeconomic stressors (Cummins 2024). In 2024, these countries received approximately $975 million in USAID disbursements that directly and indirectly supported maternal health-related services.

The remainder of this article is structured as follows. The Literature review section summarizes important studies on health expenditure, maternal mortality, and ODA volatility. The Methods section details the model and data sources. The Results section presents the main findings. The Discussion section describes interpretations and limitations. And the Conclusion section presents final thoughts.

## Literature review

A substantial body of empirical research documents an inverse relationship between public health expenditure and maternal mortality. Cross-country analyses consistently find that increased government health spending is associated with lower maternal mortality ratios, particularly in low-income settings with limited baseline service coverage.

Early work by Bokhari et al. (2007) demonstrated this association across 127 developing countries. Their analysis found that a 1% increase in per capita public health expenditure correlates with a 0.5% reduction in the MMR.

Subsequent studies focusing on sub-Saharan Africa report comparable elasticities. Nketiah-Amponsah (2019) studied 46 countries over 2000–15 and identified that a 1% increase in per capita health expenditure reduces maternal mortality by 0.35%. Arize et al. (2024) reached a nearly identical conclusion when looking at a similar country sample between 1960 and 2022: a 1% increase in per capita health expenditure leads to a reduction in maternal mortality by 0.34%. When looking at 13 countries in Southern Africa spanning from 2000 to 2022, Dinga et al. (2024) conclude that greater government health expenditure significantly reduces maternal mortality, but the impacts can be moderated by levels of external debt stock. Dieleman et al. (2016) focus on the role of donor dependency, noting that maternal health programs absorb around 10% of total development assistance for health and falter without sustained funding.

Country-specific studies across sub-Saharan Africa reach similar conclusions. In Nigeria, e.g. Akintunde et al. (2023) concludes that a 1% increase in total government health expenditure leads to a 37% lowering of maternal mortality over the long term while earlier work by Nwankwo (2018) found the same change in expenditure reduces the MMR by approximately 6 deaths per 1000 live births. In Zimbabwe, Manyika et al. (2023) estimated that a 1% increase in government health expenditure is associated with a 0.5% reduction in maternal mortality. Other studies underscore the importance of public health expenditure in ensuring the availability of essential maternal health services to reduce MMR, including in Kenya (Awe et al. 2023) and Uganda (Atuhaire et al. 2024).

Of particular relevance to the present analysis is the study by Boundioa and Thiombiano (2024), which focuses on the West African Economic and Monetary Union (WAEMU) and includes three of the countries examined in this article (Burkina Faso, Mali, and Niger). Using panel data from 2000 to 2020, they found that a 1% increase in health expenditure per capita results in a 2.8% reduction in the MMR, which is largely driven by enhanced access to skilled delivery care and emergency obstetric services. Crucially, the authors warned that sudden drops in public health investment, especially those linked to external funding shocks in fragile health environments, could quickly reverse years of progress in maternal health, a scenario directly applicable to the experience of USAID in 2025.

Recent counterfactual studies have examined the potential health effects of abrupt USAID funding withdrawal under explicit “no-substitution” assumptions. Cavalcanti et al. (2025) estimate that USAID investments prevented millions of deaths and project sizeable reversals if funding were removed without replacement. Commentaries on this work emphasize that such estimates represent short-term upper-bound effects and depend critically on assumptions about the absence of compensatory financing (Krugman et al. 2025).

Taken together, the literature indicates that maternal mortality outcomes are highly sensitive to both the level and stability of health financing. In settings with constrained domestic fiscal capacity, abrupt aid withdrawal may therefore have substantial short-term impacts.

## Materials and methods

This study examines six countries in West and Central Africa that were highly exposed to the sudden withdrawal of USAID health funding at the start of 2025. The outcome of interest is the MMR, defined as the number of maternal deaths per 100 000 live births.

This analysis employs a deterministic model linking changes in public health expenditure to changes in maternal mortality, drawing on region-specific expenditure elasticities from the literature. The model estimates short-term impacts under the assumption that lost USAID resources are not immediately offset by domestic reallocation, other donors, philanthropic financing, and/or increased household expenditure during 2025. This assumption is consistent with prior counterfactual analyses of abrupt aid withdrawal and is intended to illustrate potential near-term impacts.

Public health expenditure is defined as the sum of domestic general government health expenditure and external health expenditure. Data sources include:

- USAID funding: USAID disbursements in 2024 to five “US sectors” that directly and indirectly support maternal health services—(i) Family planning and reproductive health, (ii) Health—general, (iii) Maternal and child health, (iv) Nutrition, and (v) Protection, assistance, and solutions—are from USAID and US Department of State (2025).
- Health expenditure: The highest reported value between 2019 and 2022 for domestic general government health expenditure (GGHE-D) per capita and external health expenditure per capita (both in current US$) are from the WHO (2025b).
- Demographic: Medium and high variant estimates for total population, women in reproductive age (15–49), and crude birth rate for 2025 are from UN DESA (2024)
- People in need: The number of persons defined as in need of humanitarian support as of January 2025 is from United Nations Office for the Coordination of Humanitarian Affairs (UN OCHA) (2025).
- Maternal mortality: Medium and high variant MMRs for 2020 (the latest available) are from WHO, United Nations Children’s Fund (UNICEF), United Nations Population Fund (UNFPA), World Bank Group, and United Nations Department of Economic and Social Affairs (UNDESA) (2023).

The model specifications are described below.

### Equation 1. Per capita United States Agency for International Development funding for maternal health services to people in need

The first step is to estimate the per capita value of USAID funding that was likely to support maternal health services in 2025 among people in need from US Department of State (2025). This is calculated by adding USAID disbursements to five sectors in each country in 2024 in current US$, applying 29.8% as the estimated amount that supported people in need in 2024, and dividing by the total number of people in need as of January 2025 from UN OCHA (2025).


$$PC_{\mathrm{usaid}}=\frac{F_{\mathrm{usaid}}\times P_{\mathrm{usaid}}}{P_{\mathrm{total}}}$$

Where $F_{\mathrm{usaid}}$ is USAID disbursements to five sectors in each country in 2024 (in current US$), $P_{\mathrm{usaid}}$ is Estimated portion of USAID funding that supported people in need in 2024 (29.8%), and $P_{\mathrm{total}}$ is the total number of people in need in January 2025 (UN OCHA 2025).

### Equation 2. Impact of United States Agency for International Development funding loss on total health expenditure

The second step is to estimate the impact of the loss of USAID funding on overall health expenditure on people in need in 2025. This requires summing the per capita current US$ values of domestic general government health expenditure and external health expenditure based on WHO (2025b), then subtracting the per capita USAID funding to determine the percentage change. To prevent overestimating the spending shock, the highest reported expenditure values from 2019 to 2022 were applied.


$$\Delta PC_{\mathrm{total}}=\left(\frac{PC_{\mathrm{domestic}}+\;PC_{\mathrm{external}}-\;PC_{\mathrm{usaid}}}{PC_{\mathrm{domestic}}+\;PC_{\mathrm{external}}}\right)\;\times 100$$

Where $PC_{\mathrm{domestic}}$ is per capita domestic general government health expenditure (WHO 2025b), $PC_{\mathrm{external}}$ is per capita external health expenditure (WHO 2025b), and $PC_{\mathrm{usaid}}$ is per capita USAID funding to maternal health-related services (Equation 1).

### Equation 3. Maternal deaths without United States Agency for International Development funding cuts

The third step is to estimate the number of maternal deaths that would take place among women in need if USAID funding continued unaffected in 2025. The total number of live births is derived by multiplying the share of women in reproductive age in 2025 from UN DESA (2024) to the population in need and then applying the crude birth rate high variant estimate. The MMR high variant estimate from WHO, UNICEF, UNFPA, World Bank Group, and UN DESA (2023) is then used to determine the baseline number of maternal deaths.


$$MD_{\mathrm{baseline}}=(P_{\mathrm{total}}\times P_{\mathrm{reprod}}\times B_{\mathrm{crude}})\times \mathrm{MMR}$$

Where $P_{\mathrm{total}}$ is the total number of people in need in January 2025 (UN OCHA 2025), $P_{\mathrm{reprod}}$ is the share of women in reproductive age in 2025 (UN DESA 2024), $B_{\mathrm{crude}}$ is the crude birth rate high variant estimate in 2025 (UN DESA 2024), and *MMR* is the maternal mortality ratio high variant estimate in 2020 (WHO et al. 2023).

### Equation 4. Excess maternal deaths due to United States Agency for International Development funding cuts

The final step is to estimate the excess maternal deaths among women in need resulting from the decline in health spending due to the dismantling of USAID. This is done by applying the coefficient from Boundioa and Thiombiano (2024)—which quantifies the relationship between a percentage point change in public health expenditure and a change in MMR among countries in the WAEMU [−0.078*** (***Indicates a highly statistically significant result where the *P*–value is less than .001)]—to Equation 2 and comparing the projected maternal deaths to the results in Equation 3.


$$MD_{\mathrm{excess}}=(((\Delta PC_{\mathrm{total}}\;\times \;\mathrm{Coef})+\mathrm{MM}R_{\mathrm{baseline}})\times B_{\mathrm{total}})-MD_{\mathrm{baseline}}$$

Where $\Delta PC_{\mathrm{total}}$ is the percentage change in health expenditure (Equation 2), Coef is −0.078 (based on Boundioa and Thiombiano 2024), $\mathrm{MM}R_{\mathrm{baseline}}$ is the maternal mortality ratio (Equation 3), $MD_{\mathrm{baseline}}$ is maternal deaths estimated (Equation 3), and $B_{\mathrm{total}}$ is the total number of live births among women in need (Equation 3).

## Results

**Figure 1:**
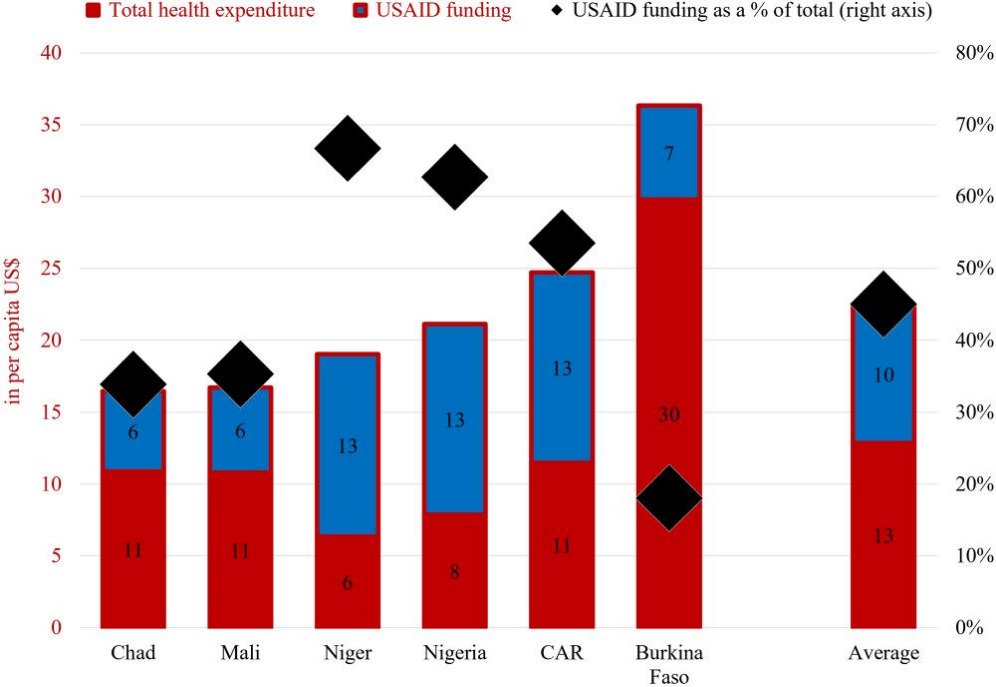
Total health expenditure (government and external) and USAID funding for maternal health-related services for persons in need in six countries in West and Central Africa, 2025 projections (in per capita US$). Source: Model outputs.

The loss of USAID funding is projected to substantially reduce health expenditure among populations in need across all six countries. On average, the reduction amounts to approximately US$13 per person, corresponding to a 45% decline in total health spending available to these populations (Fig. 1). The estimated reduction ranges from just under 20% in Burkina Faso to around 35% in Chad and Mali and above 50% in CAR, Niger, and Nigeria.

**Figure 2:**
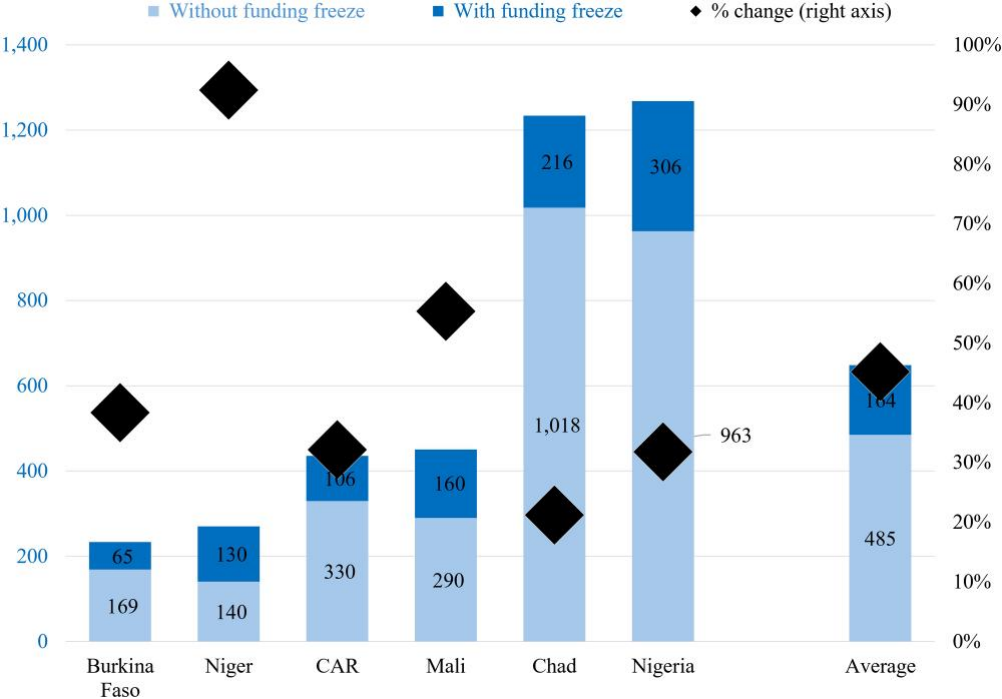
Estimated excess maternal deaths due to the ending of USAID funds in six countries in West and Central Africa in 2025 (in # of maternal deaths and as a % change). Source: Model outputs.

**Figure 3:**
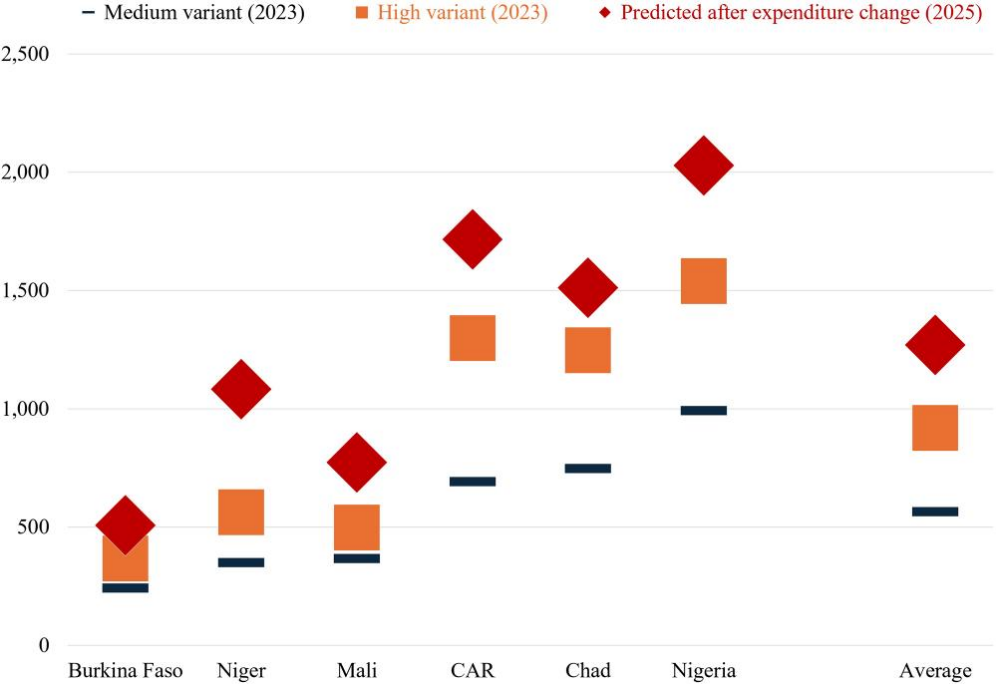
MMRs in six countries in West and Central Africa under different scenarios. Source: Model outputs.

When focusing on the impacts of reduced spending on women in need who were likely to give birth during 2025, the model predicts that maternal deaths would increase by 45%, on average (Fig. 2). The contraction in expenditure is associated with an increase in MMRs from ∼920 to 1270 deaths per 100 000 live births, on average (Fig. 3). In absolute terms, maternal deaths are estimated to rise from a baseline of ∼2900 to nearly 3900 in 2025, yielding around 1000 additional deaths.

The magnitude of impact varies across countries. Niger is predicted to experience the largest proportional increase in maternal mortality (an increase of more than 90%), while Nigeria accounts for the largest number of excess deaths (more than 300).

### Statistical significance and confidence intervals

As described in Methods section, the projections are based on an elasticity coefficient of −0.078 from the literature. To assess the robustness of these estimates, a sensitivity analysis was conducted using elasticity coefficients ranging from −0.06 to −0.10. The resulting variations in projected MMRs were within acceptable margins, reinforcing the reliability of the findings. 95% confidence intervals were also calculated for the projected increases in MMRs, considering potential variability in health expenditure reductions and elasticity estimates. The confidence intervals indicate that the projected increases are statistically significant (*P* < .05) across all countries.

## Discussion

### Alignment with previous evidence

This analysis provides quantitative estimates of the short-term maternal mortality implications of abrupt ODA reductions for health systems in six countries in West and Central Africa. Under a scenario in which lost resources are not rapidly replaced, the model suggests a 45% average increase in maternal mortality among populations already facing humanitarian vulnerability. The magnitude and direction of the estimates are consistent with a long empirical tradition linking public health spending to maternal health outcomes, including Bokhari et al. Gottret (2007), Nketiah-Amponsah (2019), Arize et al. (2024), and Boundioa and Thiombiano (2024).

The results should be interpreted as conditional estimates rather than forecasts. As highlighted in recent literature, realized impacts depend on the timing and scale of compensatory responses by governments, multilateral institutions, and other donors (Cavalcanti et al. 2025, Krugman et al. 2025).

### Heterogeneity across countries

Cross-country heterogeneity reflects differences in baseline fiscal capacity, donor dependence, and service coverage. Countries with relatively larger domestic health budgets experience smaller proportional shocks (e.g. Burkina Faso), while highly aid-dependent systems face larger immediate effects (e.g. Niger, Nigeria). These differences echo Dinga et al. (2024), who showed that the fiscal space created by domestic revenue—or constrained by debt—modulates the impact of health spending cuts. They also highlight the role of baseline service coverage: where institutional deliveries and emergency obstetric capacity are already sparse, every marginal dollar lost has a larger marginal effect on survival.

### Policy implications

This study has three important policy implications. First is emergency bridge financing. The speed and scale of the projected increase in maternal deaths requires an urgent humanitarian response. Multilateral actors (the World Bank’s Global Financing Facility, the Global Fund, UNFPA, etc.) could deploy contingent credit lines or catalytic grants to sustain essential maternal health services while domestic authorities and donors negotiate longer-term solutions.

Second is domestic resource mobilization. Ultimately, reliance on volatile external funds remains a structural vulnerability. Expanding fiscal space available for health services can reduce exposure to future foreign shocks. Priorities include increasing the share of the national budget that is allocated to the health sector, strengthening government revenue collection capacity, exploring innovating financing instruments (e.g. health taxes, impact bonds, matching funds), and negotiating debt restructuring, including through debt-for-health swaps. The inverse association between debt stock and the effectiveness of health spending identified by Dinga et al. (2024) suggests that parallel macro-fiscal reforms are indispensable.

Third is aid predictability and exit planning. Donors are responsible for ensuring that transitions out of bilateral programs are gradual, transparent, and accompanied by investments in national systems. USAID’s abrupt funding stoppage violated established aid-effectiveness principles, especially predictability and mutual accountability.

### Limitations and future research

Several limitations warrant consideration. First, the model applies a single elasticity across countries, abstracting from potential heterogeneity in health system responses. Although sensitivity checks mitigate this concern, future work could employ country-specific elasticities or dynamic panel techniques that capture non-linearities and delayed effects.

Second, the analysis focuses on populations classified as in humanitarian need and does not capture potential spillovers to the general population. As a result, the estimates likely understate total excess deaths.

Third, the model does not incorporate potential short-term financing substitution through donor realignment, emergency budget reallocation, philanthropic responses, or increased household expenditure, all of which could partially mitigate the estimated impacts. As noted in prior literature, such substitution effects are uncertain in both magnitude and timing and are therefore excluded from the base-case scenario. Nonetheless, agent-based or microsimulation approaches could illuminate these alternative financing pathways in new studies.

Finally, inflation and exchange rate dynamics may further influence real spending levels. The model adopted the highest health-expenditure values observed between 2019 and 2022 to avoid over-estimating the shock. However, inflation and exchange rate depreciation since 2022 may have already eroded real spending, suggesting that effective resource losses—and hence adverse outcomes—could be larger. This could also be addressed in future research.

### Key implications

For policymakers: Invest in domestic health financing mechanisms and fiscal reforms to reduce reliance on unpredictable external funding. Establish contingency plans to protect maternal health services during aid disruptions.

For program managers and health planners: Prioritize maintaining essential maternal health interventions (e.g. skilled birth attendance, emergency obstetric care) when external funds contract. Develop strategies to rapidly mobilize alternative resources.

For donors and development partners: Recognize the life-threatening consequences of sudden aid withdrawals. Ensure predictable, sustained funding flows and incorporate transition and exit strategies that minimize disruption to critical health services.

## Conclusion

This study estimates the potential short-term maternal mortality implications of sudden USAID health funding withdrawal in six countries in West and Central Africa. Under an explicit assumption of no immediate resource substitution, the analysis projects an average 45% increase in maternal deaths among populations in humanitarian need in 2025, corresponding to ∼1000 additional deaths beyond an estimated baseline of 2900.

The findings highlight the sensitivity of maternal health outcomes to financing continuity in fragile health systems. While actual outcomes will depend on subsequent policy and financing responses, the analysis provides evidence relevant to discussions on aid predictability, transition planning, and risk management in global health financing.

## Supplementary Material

czag034_Supplementary_Data

## Data Availability

The data underlying this article are available at *Health Policy and Planning* online.

## Reflexivity statement

As the sole author of this study, I acknowledge my professional position as a staff member of the United Nations Population Fund (UNFPA), West and Central Africa Regional Office. My role includes supporting countries in the region to strengthen health systems and improve maternal health outcomes. This positionality has informed my interest in examining the consequences of large-scale funding withdrawals for maternal mortality. While my affiliation has provided access to relevant data sources and contextual knowledge, I have taken care to maintain analytic neutrality and transparency throughout the research process.

I recognize that my professional commitment to reproductive health and development assistance may shape my interpretation of the findings, particularly regarding the urgency of policy responses to mitigate adverse impacts. To address potential biases, I have relied on publicly available data, peer-reviewed evidence, and clearly documented modeling assumptions. The views expressed in this paper are those of the author alone and do not necessarily reflect the official policies or positions of UNFPA or any other organization.
